# Synergies, tensions and challenges in HIV prevention, treatment and cure research: exploratory conversations with HIV experts in South Africa

**DOI:** 10.1186/s12910-016-0109-1

**Published:** 2016-04-30

**Authors:** Keymanthri Moodley, Theresa Rossouw, Ciara Staunton, Christopher J. Colvin

**Affiliations:** Centre for Medical Ethics and Law, Stellenbosch University, Tygerberg, South Africa; Departments of Immunology and Family Medicine, University of Pretoria, Pretoria, South Africa; School of Public Health and Family Medicine, University of Cape Town, Cape Town, South Africa; Centre for Medical Ethics and Law, Department of Medicine, Faculty of Medicine and Health Sciences, Stellenbosch University, PO Box 19063, Francie van Zijl Drive, Tygerberg, 7505 South Africa

## Abstract

**Background:**

The ethical concerns associated with HIV prevention and treatment research have been widely explored in South Africa over the past 3 decades. However, HIV cure research is relatively new to the region and significant ethical and social challenges are anticipated. There has been no published empirical enquiry in Africa into key informant perspectives on HIV cure research. Consequently, this study was conducted to gain preliminary data from South African HIV clinicians, researchers and activists.

**Methods:**

In-depth interviews were conducted on a purposive sample of fourteen key informants in South Africa. Audiotaped interviews were transcribed verbatim with concurrent thematic analysis. The perspectives of HIV clinicians, researchers and activists were captured. Analyst triangulation occurred as the data were analysed by three authors independently.

**Results:**

The rapid evolution of HIV cure research agendas was prominent with participants expressing some concern that the global North was driving the cure agenda. Participants described a symbiotic relationship between cure, treatment and prevention research necessitating collaboration. Assessing and managing knowledge and expectations around HIV cure research emerged as a central theme related to challenges to constructing ‘cure’ - how patients understand the idea of cure is important in explaining the complexity of cure research especially in the South African context where understanding of science is often challenging. Managing expectations and avoiding curative misconception will have implications for consent processes. Unique strategies in cure research could include treatment interruption, which has the potential to create therapeutic and ethical conflict and will be perceived as a significant risk. Ethical challenges in cure research will impact on informed consent and community engagement.

**Conclusions:**

It was encouraging to note the desire for synergy amongst researchers and clinicians working in the fields of prevention, treatment and cure. Translation of complex HIV cure science into lay language is critical. Moving forward, RECs must be adequately constituted with scientific expertise and community representation when reviewing cure protocols. It is hoped that knowledge and resource sharing in the context of collaboration between research scientists working in cure and those working in treatment and prevention will accelerate progress towards cure.

## Background

South African health researchers and clinicians have been actively involved in HIV research for more than three decades with a focus on prevention and treatment [1–3]. Nationally and internationally, associated ethical challenges in prevention and treatment have been widely debated [4–7]. HIV cure research is now advancing rapidly in Europe and the United States with the exploration of various scientific strategies including early treatment of acute infection, neutralizing antibodies, gene therapy, therapeutic vaccines or a combination of approaches [8, 9].

While the ethical challenges with HIV treatment and prevention research have dominated the discourse to date, there has been a growing focus on the ethical challenges of HIV cure research in recent times. Lo and Grady have highlighted key ethical issues to consider in conducting cure research including informed consent, fair selection of participants, social value, scientific validity, favourable risk-benefit ratio, independent review, respect for participants and communities and collaborative partnership [10]. Sugarman has expanded on these to include risk to partners, confidentiality and financial conflicts of interest. He adds the importance of ethical oversight by Research Ethics Committees (REC), rigorous consent processes and empirical data on consent and trial design [11]. Other authors have emphasized the conceptualisation of cure [12–14] and the challenges in ensuring adequate informed consent for HIV cure trials [15]. Given the wide range of illegitimate “cures” marketed in Africa historically, misconceptions about cure are likely to cloud understanding of cure in consent processes [16–29]. Scientifically, the distinction between a sterilizing cure (a cure that completely removes the disease from the body and renders the patient symptom free) and a functional cure (a cure that controls the symptoms without eliminating the virus) adds to the challenge of understanding cure [13, 14].

Given the complexity of HIV cure science and the wide range of stakeholders involved (HIV infected individuals, HIV clinicians, researchers, REC members, policy makers, HIV experts, health economists, health funders, the pharmaceutical industry and the media), informed consent processes for cure research in South Africa are likely to be challenging. It is therefore important to elicit multiple perspectives of a wide range of stakeholders in South Africa to inform consent processes that will be critical in the recruitment and enrolment of potential participants. There have been calls for biomedical scientists to collaborate with social scientists to develop decision-making studies as they can provide important information on perceptions of cure in real time [30]. The experiences from HIV vaccine and prevention studies highlight the importance of early stakeholder engagement in managing participant expectations as well as in helping to mitigate trial failures [31]. In HIV prevention and treatment research to date the science has sometimes progressed ahead of important social, behavioural and ethical concerns leading to unexpected or negative results [32–37]. The importance of early stakeholder engagement to explore the complex web of ethical and social concerns related to HIV cure science has therefore been emphasized [38]. To date, there have only been three empirical publications globally on stakeholder perspectives around HIV cure research: an Australian study involving patients only [39], a study from China that mainly examined the perspectives of injection drug users [40] and a South African study that elicited preliminary views of diverse healthcare providers and a few patients at an HIV research clinic in Cape Town [41].

This study reflects insights from key informants (HIV experts) who have been working in the field of HIV/AIDS in South Africa over the past three decades. It is the first empirical investigation conducted to gain preliminary data from HIV clinicians, researchers and activists to guide future phases of a larger multisite HIV cure project on related ethical and social issues where the views of other stakeholders involved in HIV cure research, including HIV-infected individuals, will be elicited.

## Methods

The perspectives of three groups of participants—researchers working in treatment and prevention, HIV clinicians and HIV activists—were elicited. The methodological approach adopted in this study is based in constructivist theory [42]. We conducted in-depth interviews with 14 South African stakeholders, including HIV clinicians, researchers, policymakers and activists in Cape Town, Johannesburg, Pretoria and Durban (South Africa) after obtaining informed consent. Interviews lasted 60 min. The demographics of those interviewed are as follows:

**Table:** 

**Key Informant**	**Profession**	**Gender**	**Sector**
Key informant A	Researcher	Female	Public
Key informant B	Clinician	Male	Private
Key informant C	Activist	Male	N/A
Key informant D	Policymaker	Male	Public
Key informant E	Researcher	Female	Public
Key informant F	Researcher	Male	Public
Key informant G	Researcher	Male	Public
Key informant H	Researcher & clinician	Male	Public
Key informant I	Researcher	Female	Public
Key informant J	Researcher & clinician	Female	Public
Key informant K	Researcher & clinician	Male	Public
Key informant L	Researcher	Male	Public
Key informant M	Social scientist	Male	Public
Key informant N	Social scientist	Male	Public

Recorded interviews were transcribed verbatim with concurrent thematic analysis. In addition, both interviewers (CC and TR) and the principal investigator (KM) read through the transcripts to extract themes (analyst triangulation). The data were analysed by three authors independently and then integrated via discussion. This study was approved by the Research Ethics Committees at Stellenbosch University (N13/05/063), the University of Pretoria (29/2015) and the University of Cape Town (761/2014).

## Results

The findings below synthesize key informant views on the important social and ethical considerations related to HIV cure research in South Africa. They are organized into five sections, each addressing a different dimension of cure research.

### Evolution and drivers of HIV cure research

An important part of the broader social and ethical context for HIV cure research involves the organization, funding and governance of this area of research, from global through to local levels. There was broad agreement among our participants that cure research is currently driven by Northern funders and researchers, largely as a result of stronger capacity, more resources, and better laboratory infrastructure required for the current forms of cure research. Research on HIV cure is only now getting underway in South Africa and appears to be driven largely by work in virology and immunology as well as some early efforts to test the effectiveness of early infant antiretroviral treatment (ART).

With respect to the funding sources for HIV cure research, participants reported little investment at this stage by the private sector (whether pharmaceutical companies or private biomedical research institutes). Instead, they indicated that the National Institutes of Health and philanthropic foundations in the global North were the main actors currently funding this stream of research.

*“Among others it will be mostly NIH in the US and … maybe even the Gates foundation ….”*

Participants noted that the total amounts of funding for cure research were still quite small in comparison to research funding for HIV prevention and treatment research. There was some concern, though, that most of the funding, and thus most of the research direction- and priority-setting, was being driven by American and European researchers and funders.

Participants, however, did not express specific concerns that this was steering cure research in one direction or another. While the general sense was that the lack of close pharmaceutical industry involvement at this early stage of cure research was probably appropriate, one participant noted that a weakness in much of the biomedical prevention research for HIV (e.g. microbicide gels) is that it often took place without substantive involvement of industry, and this in turn represented a threat to quick and sustainable scale-up in the event that effective solutions were found.

Despite the many possible paths to either a functional or sterilizing cure, most participants described HIV cure research as a fairly ‘unified field’ with little direct competition for funding and few serious debates over the most promising directions for research. Several argued that this was largely a result of the newness of the field and the limited size of available funding. There was a consensus among the researchers we interviewed that understanding the characteristics of the HIV ‘reservoirs’ in the body, as well as the ways they related to both latent (whereby the virus remains in the resting CD4+ T cells) and active infection, was a key research priority [43].

The role of the International AIDS Society (IAS) and its HIV Cure Working Group was noted by several participants as being a key shaper and driver of the field of HIV cure research. None of the participants raised concerns about the role of the IAS except to note that again, it was an organization that, in their view, had an over-representation of Northern actors and thus risked privileging their agendas and perspectives.

### HIV Cure research and the broader research and practice context

Part of understanding the social and ethical context for HIV cure research involves also considering how cure research is situated within broader research and practice contexts. For example, we asked participants about how they understood the relationships—and potential synergies and tensions—between HIV cure research and other forms of HIV research such as vaccine development or treatment research. There was a consensus among the researchers that cure research built on existing knowledge bases but that it also in turn extended knowledge in these other fields of inquiry. One participant noted the ways in which early cure research work on HIV reservoirs (“a cell type or anatomical site that allows persistence of replication-competent HIV-1 for long periods of time in patients on optimal HAART regimens”) had provided important insights for vaccine researchers [43].

*“I do think focusing on cure does assist us in understanding the cellular machinery … and certainly the vaccine people are looking at all of this with a huge amount of interest… how do you get the virus out of the cell, what is the immunology that controls things…” .*

At the same time, there was excitement that research into the HIV reservoir and other novel aspects of cure research (such as therapeutic vaccines, broadly neutralizing antibodies, and stem cells) promised to deliver new basic science insights as well. There was a sense overall that cure research would bring wider scientific benefits even if a cure proved elusive in the long run:

*“I can only see positive things, such as capacity building… the establishment of a gene therapy platform that will be useful for other purposes going forward.”*

We also asked participants about how the ‘cure agenda’ fits into the current landscape of treatment and prevention programming. We were interested in understanding if they anticipated conflicts around funding and prioritization within health service and research policies and budgets. Again, as noted above, there was a widely shared sense that given its current small scale, cure research was not yet perceived as a threat to the recognition and funding for treatment and prevention. Several participants did assert, however, that decisions around funding and priorities would always be difficult and involve trade-offs and a balancing of priorities.

*“So long as money from implementation is not diverted”*

*“Yes, well I think the overlapping concern could be that it will take money away from other…important research and programmatic areas… I think that’s an ongoing debate in the field*”

Several participants noted, though, that regardless of the potential tensions between prevention, treatment and cure agendas, it was critical to also think about prevention, treatment and cure as always existing in relation to each other. Two points were made in this vein. First, there is a complex tension between the three domains right now. HIV treatment has been remarkably effective but is of increasingly uncertain sustainability especially in light of the recent Strategic Timing of Anti-Retroviral Treatment (START) trial that recommends antiretroviral treatment for all HIV infected persons regardless of CD4 count [44]. Prevention is thus increasingly recognized as critical in the long run for turning the tide but current behavioural and biomedical interventions to reduce incidence have been promising on paper but disappointing in practice.

*“The chances are though, you know, it’s [a cure is] going to be expensive initially, it’s probably going to be relatively complicated, it’s probably going to have some side effects. So my sense is not to get the virus in the first place… You know what we know* via *the sciences that there is a lot of damage done in the first few weeks of the infection… I think it is difficult to imagine that getting a cure will outweigh prevention in the first place.”*

There was a consensus that cure was therefore a critical goal to integrate into the HIV landscape but that a safe, effective and affordable cure was also likely to be a very long way off.

Second, several participants noted that even if such a cure were to be developed, it is very likely that the need for all three interventions—prevention, treatment and cure—would persist into the future.

*“In terms of the HIV research, to a large extent, what we need to be doing is buckling down, finding people who are HIV-infected and doing what we can to ensure they can take their medicines for the rest of their lives. … We’ve got good enough medicines. If we could find everyone and treat everybody, we would be able to start seeing a decline in incidence… Research in HIV is slowing down, because it is now implementation we need to be doing.”*

Just as for cancer or TB or any number of other diseases, an HIV cure was not seen as a replacement for treatment and prevention programs but rather as an adjunct.

### Assessing and managing knowledge and expectations around HIV cure research

When asked to assess what individual and community expectations might be around HIV cure research, and how these might be managed, most of our participants initially pointed to widespread enthusiasm about the possibility of a cure, an interest that was generally stoked by media accounts of widely-known cases such as the ‘Berlin patient’ or the ‘Mississippi baby’. When asked in more detail, however, how they thought patients and communities would respond to some specific cure research scenarios, the picture they painted was more mixed. They argued that cure research would likely focus on recruiting people with HIV infection and thus people with an interest in making use of highly effective ART services. They felt that some people who were stable on treatment and virally suppressed may be reluctant to tamper with this success for research studies unlikely to benefit them.

*“If it’s not broken, why fix it?”*

*“People just need to understand that they are taking a significant risk. If they stop it [antiretroviral treatment], the virus may come back and do harm.”*

Although HIV experts expressed concern over the possible reluctance of participants to risk interrupting treatment that is working, this concern was raised by patients themselves as well as healthcare workers in an earlier study [42]. We believe that this is a valid concern.

There was also concern about the knowledge of and counselling required for participants in order to conduct this kind of research ethically. There was consensus that medical research like this always requires careful preparation and engagement at both individual and community levels, and that doing this well was complex but possible. Some difference of opinion emerged, however, about how easy this process of preparation might be. Clinical trialists working in well-resourced and well-managed research sites were generally confident and described this kind of engagement, education and counselling as routine.

*“You know good community engagement means that you understand your community you understand who your stake holders are and who your gate keepers are and that you engage in the continuous process and so, it’s community awareness, it’s community mobilization, it’s community engagement, and it’s community presentation and then it’s dissemination of information.”*

Activists, social scientists and doctors working in the public sector were, however, less confident that this could so easily be achieved without careful planning and adequate resourcing and oversight.

*“Which is why I think that the Community Advisory Board; because we really do try and draw from broad spectrum of people within the communities that we serve, would be a safer way of being able to communicate the message. Because we would start off with science based education on why cure is so difficult; and what the issues are. And then you know let them disseminate within their own communities.”*

*“I think it’s really understanding … what the intention is; or the goal of the study is, if that is explained well and …as well as possible so it shouldn’t be a rushed process and, and luckily many times there are communities where this is done and they deliberately find a systematic way of engaging with communities to prepare them before the treatment starts so if that is done that could be important in getting support from communities”.*

One particular concern shared by all, however, was around the notion of the reservoir and ‘latent’ HIV infection and the potential difficulty people might face in understanding these concepts and the ways research studies were investigating them. The fact that the HI virus can persist within the body, and even be integrated into the genomes of individual cells, has not been part of the widespread HIV awareness campaigns in South Africa, and is not a widely known medical fact. We believe that lay explanations of the notion of reservoirs would be critical in consent documents and processes.

Similarly, several participants noted that it was critical to avoid the therapeutic misconception in cure research and to also, more generally, prepare research participants as well as research staff and affected communities for the inevitability of frequent failure and even the potential for harm. Participants wanted to ensure that the current enthusiasm around cure research, and the recent successes of ART research and services, did not lull those involved into a false sense of confidence.

*“The last thing you want to do is for someone to trust in something that has not yet been proven.”*

*“From a psychological point of view, I would say that the biggest risk that we face is raising people’s hope and then not being able to meet that, so that we create false expectations that we can’t meet and people become disillusioned”*

Finally, an important difference in the social and ethical dimensions of cure research, as opposed to treatment and prevention research, emerged in participants’ recognition during these interviews that activists, communities, and civil society groups have had very little involvement so far in developing, driving and shaping the cure research agenda. This is in stark contrast to the unique and profound ways in which HIV treatment and prevention research included the voices of people living with HIV, albeit sometimes reluctantly, over the last three decades [45]. Participants expressed some ideas as to why this difference might be—specifically relating to the normalisation of HIV—but there was also some concern that this represented a potential threat to the ethical conduct of cure research in the long run.

### Challenges to constructing ‘cure’

All of our conversations with participants around HIV cure research included reflections, often extended ones, on the complexity and instability of the concept of ‘cure’:

*“What does a cure mean? Does it mean that I’m free of the disease and I never have to take pills; and I never have to go and see the doctor ever again? Or does it mean that somehow I’m controlling the virus? I think that that might be one of the issues where we differ from what patients may think the cure is going to mean.”*

There were a number of ways in which participants tried to capture these nuances. We have synthesized these under five main sub-themes.

#### A spectrum of cure(s)

Participants all noted the critical distinction made in the current HIV cure discourse between ‘sterilizing’ and ‘functional’ cures, a distinction that was described by several of the researchers as a new and helpful way to think more complexly about cure. Some stated that they had not taken cure research seriously as a fruitful research avenue until they were introduced to the idea of a functional cure that would suppress replication of the virus over the long term without fully eliminating it from the body:

*“For me, ‘functional cure’ was a transition in the way I started to think about the value of cure research”.*

Once they had accepted the notion of a ‘functional’ cure for HIV, however, several participants went on to argue that medium- to long-term viral suppression without ART but following an intensive period of ART (with treatment re-initiation after recurrence) could also be considered a form of serial functional cure. There was even a suggestion by one participant that one could see standard ART itself as a cure, in that it effectively suppressed the virus and allowed the immune system to reconstitute itself. He added that this was, pragmatically speaking, one form of cure that many people may be reluctant to give up for participating in research with uncertain personal benefits.

#### A long and uncertain pathway to cure

There was also recognition that cure research—involving whatever form of cure one might imagine—would almost inevitably have to pass through many years of weakly and partially effective cures, unstable cures, harmful cures, and cures that continued to result in some degree of pathology (e.g. functional cures that didn’t fully suppress chronic inflammatory responses). All of these constructions of cure challenged the simple, conventional notions of cure as something that simply eliminates the infection from the body.

There was a related concern expressed by several participants about the labelling of research along this pathway as ‘cure’ research. They thought that it might be important to avoid mention of the term ‘cure’ when possible and use more limited and specific concepts such as remission, suppression, and control.

*“For patients there are various specific connotations, meaning the virus is gone. The differentiation between functional cures and eradication cures and all the rest of it, I would worry about using that language. I think that people understand control better.”*

*“I think that I might consider dropping the word cure and changing it to remission, which is a kind of word that we use in cancers to say ‘I can’t find your sickness now but it doesn’t mean that I’m never going to’ and you would have to have follow up to make sure. Definitely people think that cure means that it is gone”.*

#### The long time horizon of ‘cure’

In contrast to a simple notion of cure, concepts like remission and suppression also entail an anticipation of the recurrence of disease. Participants noted that unlike prevention and treatment, the success of which could often be measured in days or weeks, the time horizon for the establishment of cure was potentially very long. Borrowing again from the concept of ‘remission’, some participants mentioned the ‘five-year rule’ of remission and noted that it was vital to remember that unless a fairly stable and certain cure could be developed, the concept—and the lived experience—of ‘cure’ was likely to be dependent on ongoing confirmation of cure:

*“I’ve heard people with cancer talk about their remission as a cure; and yet I would say strictly speaking doctors would say ‘in remission’ means we cannot guarantee that it won’t come back.”*

For most people, this would play out over a long, possibly open-ended time horizon and require constant monitoring.

#### Lay versions of cure

Participants also noted the ways that the notion of cure could be understood quite differently outside of biomedical contexts. They described the differing cultural constructions of cure that are often at stake in South African (and many other) contexts. These alternative notions of cure included both naturalistic forms of cure (such as local herbal remedies and tonics) as well as more supernatural forms of cure that involved spiritual modes of healing. A couple of participants noted, in addition, that while one could easily find all manner of local ‘lay’ cures being offered within these context of traditional, alternative and complementary medicine, pharmaceutical companies were also engaging in this discourse. One participant described the ways in which one drug company was promoting its latest drug’s ability to tackle the HIV reservoir, an implicit gesture towards the cure discourse, a claim that this participant argued was unsupported by the evidence:

*“[A cure] has to be marketed honestly. You know they have these newer combinations now which talk about helping reduce the reservoirs of virus……we just don’t know and there’s not enough evidence”*

The multiple and sometimes self-interested meanings of cure, and the ability of a wide range of actors to transform and use it to their own ends, were noted as an ethical concern in research billed as ‘cure research’.

#### Cures out of reach

Finally, a couple of participants noted that a cure that was not affordable or accessible was not really a cure at all and that researchers and policymakers must be vigilant in ensuring cure research takes issues of equity in the distribution of risks and benefits of research seriously.

*“… obviously it’s got to be a process that if we do it to one we can ultimately do it to all; so things like bone marrow transplants, very invasive things like that are never going to be the solution, because we can’t ablate everybody’s bone marrow,… so it would not be accessible to the… ordinary person in the street* …” *.*

### Ethics in HIV cure research

While all of the above themes raise issues that have a bearing in some way on research ethics in HIV cure research, we also asked participants about specific research ethics issues related to HIV cure research. Many of the ethical concerns they raised, while relevant to cure research, were issues that are also applicable to HIV and medical research more generally. They pointed to the potentially significant and often unknown harms of new drugs and medical procedures. They asked questions about whether, how, and when to include children and other vulnerable groups in research. They argued for the importance of operational and community-oriented research to support implementation of potential cure modalities. They were concerned about the potential inequity of the distribution of cure research risks and benefits (especially between the global North and South). And as noted above, they highlighted the difficulty of negotiating truly informed consent in medical research, especially in contexts with low levels of health literacy and/or high levels of distrust in biomedicine and academic research as is often the case in South Africa.

We were especially interested, however, in finding out what participants considered to be the ethical issues that were either unique to, intensified by, or complicated by cure research in particular. Four key issues emerged:

#### Managing potentially unrealistic expectations and avoiding curative misconception

Like therapeutic misconception that often arises in the context of treatment trials and other research, curative misconception could occur in HIV cure trials. Participants noted the critical importance of avoiding curative misconception as ‘cure’ research is at a very early stage of research (which means that almost no one should have any expectation of individual benefit, unlike in later phase trials):

“*I think it’s got to be explained really carefully that there’s no guarantee. The place we have seen a parallel to this is in prevention research. ‘So we’re giving you a tablet…but…it’s a placebo controlled trial so we’ve got no way of guaranteeing that this is going to work or even that you’re on an active drug. So you’ve got to put in all the other safeguards of safer sexual practices’. Because the last thing you want to do is [for] someone to trust in something that hasn’t yet been proven”*

Some participants highlighted the potential risk of cancer, damage to the immune system, activating previously dormant virus and other unknowns.

*“People need to understand they are taking a significant risk”.*

Another aspect is behavioural disinhibition or risk compensation, which has parallels in prevention research.

*“In terms of risk compensation, that will happen whenever there is any new technology. Well, if it works, it is extremely difficult to get people to believe that, you know they’ve gone through some procedure, plus you are still insisting that they use condoms, for example. In a way it does create what we as psychologists call ‘cognitive dissonance’ – these conflicting ideas.*

#### Psychological toll of long-term medical surveillance

Several participants described in some detail the psychological toll of long-term medical surveillance—for recurrence of an illness, for a diagnosis, or for treatment failure—and argued that cure research both heightened the stakes involved while also lengthening the time horizon required by research.

*“Failure might be after 5 years of suppression…That anxiety of coming in all the time for your blood tests and monitoring…it will be a very interesting period for people who have to go through that. The emotional highs and lows will be rollercoaster.”*

#### Potential difficulties in understanding concepts of latent infection and the HIV reservoir

As we have noted earlier, there were concerns about the potential difficulties in understanding concepts of latent HIV infection and the HIV reservoir, and suggestions that 1) research participants be well prepared, and properly counselled and supported during the study, and 2) studies use more limited and specific language to frame their research objectives:

*“The distinction between a functional cure and eradication is difficult and the word “****control****” is probably better. When researchers talk about cure, participants might think that the virus is gone. This would be bad. For instance, with herpes and chickenpox we don’t use the word cure.”*

#### Treatment interruption

Finally, a widely shared concern among the participants was the likelihood that HIV cure research would require people who are stable on ART to stop their treatment as part of HIV cure research, and then resume it at a later date:

“*I’m sure people who are living with HIV…would be interested in trying out something but…the question is…whether during the time they have to stop taking their ARVs – that might be a bit of a concern. I’m actually trying to figure out how one would do it …ethically because we know ARVs…enable one to control or manage the condition.”*

There were concerns that this might interrupt established routines of treatment adherence, allow time for the HIV reservoirs to grow in size, increase the risk of resistance and onward transmission, and possibly cause unforeseen medical complications such as a higher incidence of cardiovascular disease as observed in the SMART trial. Similar concerns around treatment interruption were raised by healthcare providers and patients in an earlier study [42] and based on our collective experience in HIV research in South Africa, we believe these concerns will be valid in future cure research.

## Discussion

HIV cure research is complex. Consequently scientific, ethical and social challenges are anticipated. From the perspective of HIV researchers, clinicians and activists in this study it was evident that concerns filtered down from broad policy issues to practical concerns that will impact more directly on patients and potential research participants. At a macro-level two major concerns exist – the power asymmetry between the global North and South and the relationship of cure to treatment and prevention research.

It is clear that concerns exist over the current predominance of the global North in setting the cure agenda, the disproportionate funding available to resource rich settings and the lack of scalability of current cure strategies for Africa [46, 47]. This concern about the unequal relationship is a long-standing ethical and political concern with respect to HIV research and intervention in resource depleted settings. The absence of the voice of the community in setting cure agendas is also palpable. Campbell et al. have previously commented that “health-related experiences and worldviews of grassroots communities….were subordinated to the imperatives of international experts and funders using western, individual-focused biomedical and behavioural models” in the context of HIV research [48]. Power asymmetries clearly play a role at multiple levels. Scalability is particularly important in the African setting where the burden of HIV is enormous. This speaks directly to ethical concerns around equity in the global distribution of risks and benefits of HIV cure research. It is essential for any cure strategy to be translated into “locally and culturally appropriate discourses and practices”, for local capacity to be built to sustain cure interventions beyond funding periods and that health systems are strengthened in resource poor settings [47].

The synergies and tensions that exist between cure, treatment and prevention were regarded as a second broad concern. Although researchers working in cure and prevention acknowledge the scientific value that cure is adding to these fields, there was some concern that resources could be diverted from treatment and prevention to cure, a concern raised by others several years ago [8]. While this is not perceived as a threat at present, it is something to consider for the future.

Moving down the ‘concern funnel’, patient understanding and expectations were highlighted as important considerations in the consent process and the decision to enrol in future cure trials. Similar challenges in obtaining informed consent for future HIV cure trials in South Africa have been raised by others [15]. In particular, explaining the concepts of latent infection and “reservoirs” were perceived to be a major challenge in future consent processes. This is linked to notions of cure, which might differ considerably from medical scientists, who might easily distinguish between functional and sterilizing cures, to patients who may conceive of cure very differently. Research into local notions of cure, for example, has highlighted the fact that for many people in the South African context, cure is often equated with the resolution of symptoms rather than with the elimination of viral particles from the body [48].

A note of caution has therefore been advised over the use of the word cure. HIV clinicians and researchers in this study discussed the construction of cure as problematic on various levels. We concur. In response to similar concerns, several authors have suggested alternatives such as “remission” or “experiment” [12–14]. The latter term in particular has been suggested to ensure that participants do not overestimate the benefits of the trial. Several authors distinguish between therapeutic misconception [49, 50] or curative misconception [13] and therapeutic misestimation [51]. Therapeutic misestimation occurs when participants overestimate the benefits or under estimate the risks and this can be tolerated, but a curative misconception would involve the participant believing that s/he will be cured with the research. This fundamentally impacts a participant’s decision to enrol in a trial and s/he may not be making a truly autonomous decision. This is particularly important if a cure trial design includes treatment interruption [52–54]. Under these circumstances, it is important that research participants fully appreciate the risks including drug resistance, the risk of transmission to sexual partners, severe primary infection-like symptoms, reduced CD4 count and increased risk of cardiovascular events and AIDS-related events [55–58]. Given the history of illegitimate HIV “cures” in South Africa and the complexity of HIV cure language and concepts, we support the concern expressed by HIV experts in South Africa regarding consent processes for future cure research. Nothing short of an intense community engagement process with attention to science translation will facilitate authentic consent processes in this new field of HIV research.

Ultimately REC members are charged with oversight of consent processes and must therefore be cognisant of the challenges in understanding the complexity of cure science. Consequently RECs must be adequately constituted with appropriate scientific and community expertise [11]. Both RECs and researchers have an obligation to ensure that the information in consent forms does not raise undue expectations.

This study sought to establish the views of prominent HIV experts in South Africa working in the field of research, treatment or activism. There are, however, many other HIV clinicians working in public health clinics in South Africa and some of their views must also be captured. This will be attempted in the next phase of this study where the views of other stakeholders, including HIV infected patients will be explored. Likewise the views of policy makers, private health care funders, the pharmaceutical industry, traditional healers, health economists, patients, religious leaders and the media remain to be explored. The need for early stakeholder engagement has been echoed by others [40, 52].

Future research opportunities exist to explore international collaborations dealing with cure and how research agendas are being set. South African researchers and clinicians have a wealth of experience and expertise in HIV research and care. Consequently, they ought to be playing a greater role in setting cure agendas.

## Conclusions

A holistic approach integrating biomedical treatment, prevention and cure research is critical to control the HIV epidemic. It was encouraging to note the desire for synergy in these three domains amongst researchers and clinicians. However, the social and ethical dimensions of cure must also taken into account. HIV experts in this study appropriately raised concerns about the dominance of the global North in driving the cure research agenda and the absence of civil society voices in shaping cure research priorities and future access. The call for global equity in driving the HIV cure research agenda is a call to ensure a fair distribution of risks and benefits between the global North and South. Scientists and clinicians raised valid concerns about the various constructs of cure and translation of complex HIV cure science into lay language to enhance consent processes. This is an important consideration in South Africa where consent processes are challenging for less complex research. Likewise, it is an ethical obligation for RECs to be adequately constituted with scientific expertise and community representation when reviewing cure protocols. It is hoped that knowledge and resource sharing in the context of collaboration between research scientists working in cure and those working in treatment and prevention will accelerate progress towards cure.

### Availability of data and materials

For reasons of confidentiality we are unable to share our interview transcripts. There are a limited number of HIV experts in SA and the anonymity of this study can be compromised by release of transcripts.
